# The β-triketone, nitisinone, kills insecticide-resistant mosquitoes through cuticular uptake

**DOI:** 10.1186/s13071-025-06939-0

**Published:** 2025-07-31

**Authors:** Zachary Thomas Stavrou-Dowd, George Parsons, Clair Rose, Faye Brown, Rosemary Susan Lees, Álvaro Acosta-Serrano, Lee Rafuse Haines

**Affiliations:** 1 Department of Vector Biology, Liverpool School of Tropical Medicine, Pembroke Place, Liverpool, L3 5QA UK; 2 Present Address: Institute for Cell and Molecular Biosciences, Newcastle University, Newcastle Upon Tyne, NE2 4HH UK; 3 Present Address: UK Health Security Agency, Porton, Salisbury, SP4 0JG UK; 4 Present Address: Department of Biological Sciences and Eck Institute for Global Health, University of Notre Dame, Notre Dame, IN 46556 USA

**Keywords:** Vector-borne diseases, Mosquitoes, Tarsal, 4-Hydroxyphenylpyruvate dioxygenase, Nitisinone, Insecticide resistance

## Abstract

**Background:**

Insecticide resistance in disease-transmitting arthropods of agricultural, veterinary, and public health significance poses a significant threat to vector control programs worldwide. Previous studies demonstrated that blood-feeding arthropod vectors experience high mortality when ingesting blood containing inhibitors of 4-hydroxyphenylpyruvate dioxygenase (HPPD), the second enzyme in tyrosine metabolism. This study investigated the mosquitocidal efficacy of HPPD inhibitors from the β-triketone class of herbicides against both susceptible and pyrethroid-resistant strains of three major disease vector species, including mosquitoes that transmit historical diseases such as malaria, reemerging infections such as dengue and Zika, and emerging viral threats such as Oropouche and Usutu viruses.

**Methods:**

Four HPPD inhibitors (nitisinone, mesotrione, sulcotrione, and tembotrione) were screened using glass plate tarsal bioassays at 125 mg/m^2^ against bloodfed *Anopheles gambiae* s.s. Kisumu. Nitisinone was selected for evaluation against susceptible and pyrethroid-resistant strains of *An. gambiae* s.s. Kisumu, *An. gambiae* s.l. Tiassalé 13, *An. coluzzii* VK7 2014, *Culex quinquefasciatus* Muhezha, and *Aedes aegypti* New Orleans. Mosquitocidal activity was assessed using glass plate tarsal contact bioassays, topical application assays (0.0001% to 1% w/v), and modified Centers for Disease Control and Prevention (CDC) bottle bioassays (0–30 μg per bottle). Female mosquitoes aged 3–5 days were bloodfed within 1 h before exposure. Mortality was recorded at 30 min and 24, 48, and 72 h post-exposure under controlled conditions. A total of 3 biological replicates of 30 mosquitoes per treatment were used.

**Results:**

Only nitisinone, and not mesotrione, sulcotrione, or tembotrione, exhibited significant mosquitocidal activity when bloodfed mosquitoes were exposed to treated surfaces. No significant differences in susceptibility to nitisinone were observed between insecticide-susceptible *An. gambiae* and strains harboring multiple insecticide-resistance mechanisms. The compound demonstrated consistent efficacy across all three mosquito species tested, indicating broad-spectrum activity against major disease vectors.

**Conclusions:**

This study demonstrates that nitisinone exhibits a novel mode of action distinct from current Insecticide Resistance Action Committee (IRAC) classifications by specifically targeting blood digestion processes. Its efficacy against resistant strains and potential for integration into existing vector control interventions, such as treated bednets and indoor residual spraying, highlight nitisinone as a promising candidate for expanding strategies against malaria, dengue, Zika, and other emerging viral diseases.

**Graphical Abstract:**

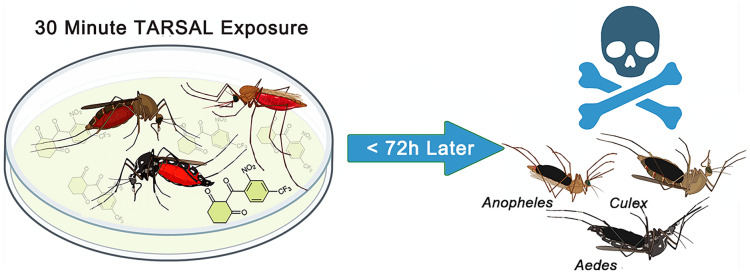

**Supplementary Information:**

The online version contains supplementary material available at 10.1186/s13071-025-06939-0.

## Background

Exposure to insecticides (by the agricultural industry or during public health campaigns for vector control) place intense selective pressure on insect populations and thereby contribute to the emergence of insecticide resistance. This compromises the efficacy of insecticide-based control strategies. Developing new or repurposed chemistries with different insecticidal modes of action is vital to combating this resistance. Pre-2017, malaria cases notably declined, with a global reduction of 18%, but 20% specifically in malaria-endemic countries in Africa. This reduction in malaria incidence is largely attributed to the expansion of key vector control interventions, notably the widespread distribution and use of long-lasting insecticidal nets (LLINs) and indoor residual spraying (IRS) [1–3].

However, a reported plateau of malaria cases since 2017 is in part due to mosquitoes’ developing resistance to insecticides. The use of IRS is predicated on the feeding behavior of the *Anopheles gambiae *sensu lato, as this species both feeds and rests indoors. When this mosquito rests on an insecticide-sprayed surface, it dies [4]. According to the World Health Organization (WHO), the most significant effect of IRS occurs after mosquitoes have fed on blood. When resting on an insecticide-treated surface post bloodmeal, a lethal insecticide dose is absorbed through their cuticle, which will immediately compromise the mosquito and prevent malaria transmission [4].

*Culex quinquefasciatus*, a global vector of several arboviral diseases, avian malaria, and lymphatic filariasis, has also developed resistance to multiple insecticides in countries spanning four continents [5–13]. Blood-feeding is highly dependent on host availability. This species feeds regularly on birds and domestic animals and less often on humans [14–18].

*Aedes aegypti* transmits many arboviruses including dengue, Zika, and Chikungunya, and is also an urban pest species. This species’ urban nature allows for unconstrained proliferation, particularly as the global population becomes more urbanized. Increasing global temperatures are predicted to further multiply its density and range [19]. For dengue virus, these factors have put more than half the global population at risk [20].

Mosquitoes, as with all hematophagous arthropods, typically ingest a large volume of blood when feeding. This volume can often exceed several times the female’s body weight in a single feeding. For example, mosquitoes, sand flies, and reduviid bugs can ingest 3–10 times their body weight in blood [21]. Mammalian blood is rich in protein and several amino acids, including tyrosine [22]. One key detoxification enzyme within the tyrosine pathway, 4-hydroxyphenylpyruvate dioxygenase (HPPD), can be blocked using commercially available HPPD-inhibiting herbicides, including members of the β-triketone family [23]. The repurposed human drug nitisinone [24, 25], a potent HPPD-inhibitor of herbicides, has been demonstrated to kill several blood-feeding arthropods when co-administered with the bloodmeal [19, 26–30]. However, the use of these inhibitors in this manner, either as ectocides or endectocides, could carry ethical concerns and a high safety criteria for implementation [31].

When used to treat tyrosinaemia type I and alkaptonuria, nitisinone demonstrates a good overall safety profile in toxicology studies. While reversible corneal effects have been reported in rats and beagle dogs at low doses, no such effects were seen in mice, rabbits, or rhesus monkeys, even at much higher doses over extended periods [32, 33]. In humans, side effects are typically linked to long-term use and elevated levels of tyrosine in the blood, which can cause temporary eye issues, and in rare cases, cognitive effects. Further attesting to its positive safety profile, it is approved for long-term use in both children and adults, including newborns. These risks are unlikely to be relevant in the short-term, nonsystemic exposures expected from public health applications [34]. Environmentally, nitisinone is chemically stable under typical storage and field conditions, showing minimal degradation even in acidic or neutral environments [35]. Its combination of low nontarget toxicity and good chemical stability makes it a strong candidate for repurposing in vector control.

When mosquitoes take a bloodmeal, their tolerance to pyrethroids naturally increases [36–40], which is crucial when selecting IRS products for population control. Both wild *Anopheline* mosquitoes in various bioassays [41] and hut trials [40], as well as lab-reared mosquitoes [37, 38, 42, 43], have shown reduced mortality after blood-feeding compared with non-bloodfed counterparts. Similarly, mosquitoes feeding through baited nets exhibit reduced mortality [39]. This phenomenon may be linked to the upregulation of enzymes involved in insecticide detoxification [42].

Interestingly, standard WHO bioassays only use sugar-fed mosquitoes to validate discriminating concentrations of insecticides, which may not be lethal to bloodfed mosquitoes [38]. This highlights the importance of considering that the effective dose may vary between bloodfed and non-bloodfed mosquitoes, thus impacting residual efficacy and selection for resistance. While discriminating doses (DDs) are typically derived from LD_99_ values in sugar-fed mosquitoes, differences in the insect’s physiological state could influence susceptibility profiles, thus suggesting the range of resistance levels might not be fully captured when only sugar-fed individuals are tested.

Here we focus on testing three mosquito species, *An. gambiae*, *Ae. aegypti*, and *C. quinquefasciatus*, in bloodfed contact assays, which mimic landing on walls and targeted for IRS. All bloodfed females are killed when exposed to surfaces coated with nitisinone, but not to other β-triketone HPPD inhibitors. By leveraging tarsal uptake of HPPD inhibitors, this approach offers a promising strategy for overcoming insecticide resistance and improving vector control. This work justifies further investigation on developing nitisinone for IRS as an alternative to current insecticidal sprays.

## Methods

### Mosquito rearing for bioassays

Three strains of *Anopheline* mosquitoes aged 3–5 days were used for the initial bioassays: *An. gambiae* s.s. Kisumu strain (insecticide susceptible), *An. gambiae* s.l. Tiassalé 13 strain (insecticide-resistant and so-called sensu lato in literature [44]), and *An. coluzzii* VK7 2014 strain (insecticide-resistant). The Kisumu strain was reared in-house at the Liverpool School of Tropical Medicine (LSTM) while adult mosquitoes of the Tiassalé 13 and VK7 2014 strains were obtained from the GLP-accredited Liverpool Insect Testing Establishment (LITE) [44]. In addition, LITE provided a pyrethroid resistant strain of *C. quinquefasciatus* (Muhezha) and an insecticide-susceptible strain of *Ae. aegypti* (New Orleans) [45].

The Tiassalé strain, originating from Côte d’Ivoire, has developed resistance to pyrethroids, dichlorodiphenyltrichloroethane (DDT), and carbamates (Table S2). The VK7 (2014) strain, originating from Burkina Faso, has high resistance intensity to pyrethroids and DDT (Table S2). Both Tiassalé 13 and VK7 2014 are maintained at LITE under deltamethrin selection pressure. Muhezha is resistant to the pyrethroids permethrin and deltamethrin (Table S2). Full resistance validation can be gained from LITE [45].

The Kisumu strain was reared following previously published methods: 26 °C ± 2 °C and 80% relative humidity ± 10%, with a 12 h:12 h light: dark cycle that includes an hour of dawn and dusk. Larvae were maintained on fish food (TetraMin^®^ tropical flakes, Blacksburg, VA). To encourage female mosquitoes to deposit eggs, mated females were fed human blood purchased from the UK’s National Blood Authority (https://www.blood.co.uk) using a Hemotek Membrane Feeding System (Hemotek Ltd., Blackburn, UK). The use of human blood in the mosquito feeding protocols was conducted under the authority of license number 12548, granted to the LSTM, Pembroke Place, Liverpool, L3 5QA, UK. This license is issued under Section 16 (2)(e)(ii) of the Human Tissue Act 2004 and held by Professor Jonathan Ball (LSTM). Adult mosquitoes were maintained on 10% sucrose solution ad libitum [44, 46]. While *C. quinquefasciatus* (Muhezha) and *Ae. aegypti* (New Orleans) mosquitoes were purchased from LITE, the rearing conditions of these species, including environmental conditions, feeding of larvae, and blood-feeding, were the same as for the *Anopheles* species, the only difference being the quantity of fish food used to feed larvae due to size differences.

### Mosquito blood-feeding for insecticide-based bioassays

Prior to conducting assays, female mosquitoes were removed from cages with males and kept in female-only cages. Female mosquitoes aged 3–5 days old were sugar-starved for 5 h (to enhance bloodmeal uptake) and then offered human blood using the Hemotek Membrane Feeding System (Hemotek Ltd., Blackburn, UK) ad libitum for 45 min. After this time, the blood was removed, and fully engorged females were gently aspirated for bioassay screening. This process was carried out for all mosquito species used. To obtain bloodfed mosquitoes within an hour of feeding, during every assay bloodfed mosquitoes were actively (gently) aspirated by mouth as they finished feeding throughout the assay so that those mosquitoes feeding first and last were used first and last, respectively. In assays using non-bloodfed mosquitoes, the same sugar starvation process was followed.

### Bioassay environmental conditions

All assays were conducted in the afternoon under controlled insectary conditions of 26 °C ± 2 °C and 80% relative humidity ± 10%. All personnel involved showered the night before and refrained from using strongly scented products to maximize blood-feeding.

### Screening of HPPD inhibitors using the glass plate tarsal bioassay

Four HPPD inhibitors were chosen for tarsal screening on the basis of previous potency as an ectocide and the availability for purchase as either commercial herbicides or human drugs (Table S1) [23, 26]. All four selected inhibitors were purchased from Merck Life Science, Gillingham, UK. All inhibitors were in powdered form and catalogue reference numbers are as follows: nitisinone PHR1731; mesotrione 33855; sulcotrione 46318; and tembotrione 32766.

**Figure 1:**
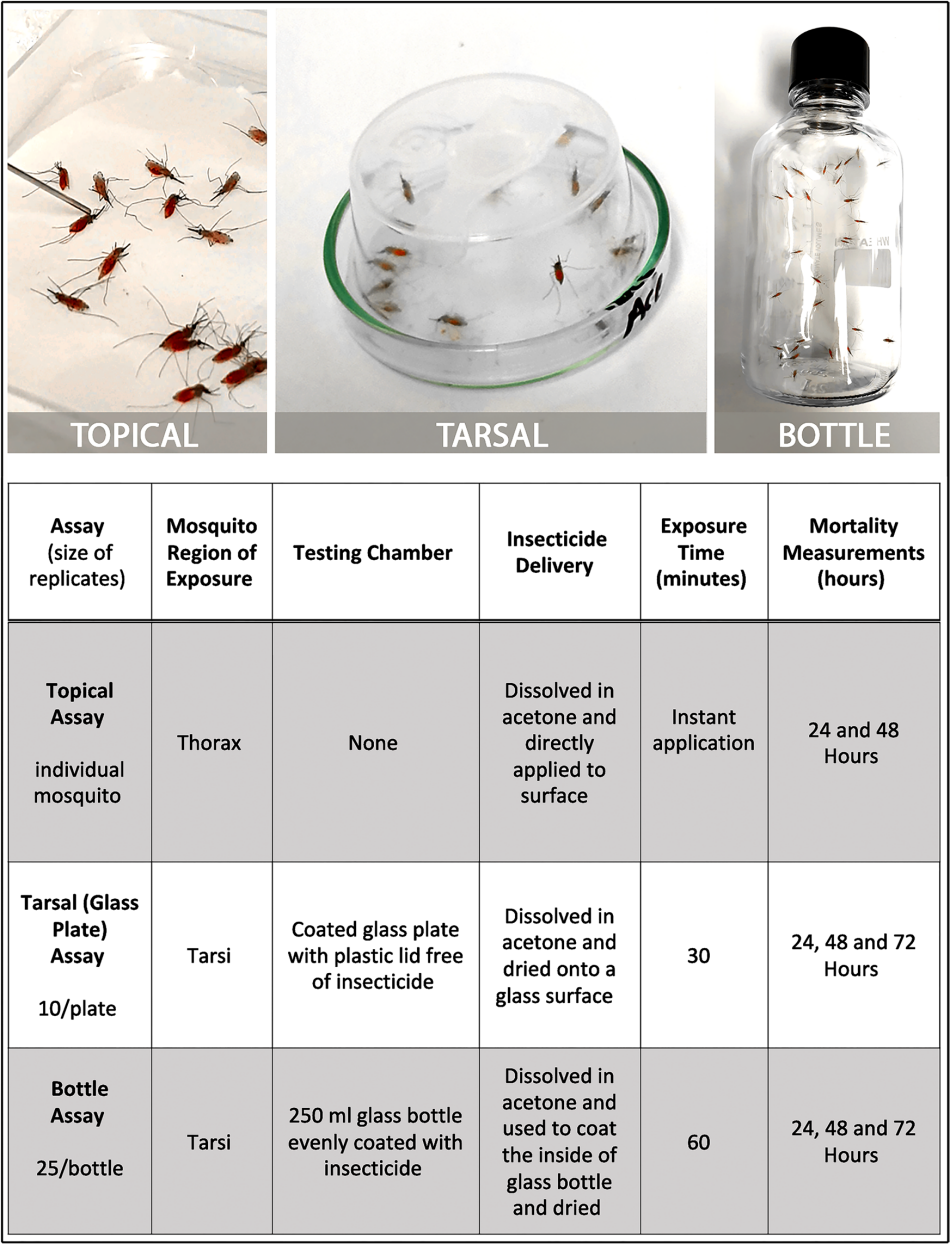
Comparison of the three assays used to assess the efficacy of nitisinone as a cuticular contact insecticide. Differences between the topical, tarsal and bottle assays, application method, insecticide delivery, and time of exposure

Using the glass plate bioassay previously described [47], a total of 3 replicates (biological) of 30 Kisumu mosquitoes (10 female mosquitoes per plate per concentration) were exposed to inhibitor-coated glass Petri dishes (VWR, Lutterworth, UK). The *Anopheles gambiae* s.s. Kisumu strain was selected because it is fully susceptible to insecticides, as the aim was to eliminate the potential confounding effects of resistance during this initial screening and focus solely on compound efficacy. Briefly, glass plates were coated with a 500 µl solution of either nitisinone, mesotrione, sulcotrione, or tembotrione dissolved in acetone (Fisher Scientific, Loughborough, UK) to a concentration of 125 mg/m^2^ (chosen as the highest dose in previous screening work [47]). This concentration was selected as an initial discriminatory screening dose to determine whether any of the HPPD inhibitors exhibited mosquitocidal activity via tarsal contact. At this early stage, the aim was not to assess field applicability or cost-effectiveness, but rather to identify any active compounds for further investigation. The solvent, acetone, was used as the negative control. Petri dishes were dried for 4 h at room temperature in a fume hood prior to use. Mosquitoes were exposed to coated plates for 30 min (Fig. 1) and then carefully removed from the testing plate to a cage using a vacuum into separate paper cups per plate. Mortality was tracked at 30 min, 24 h, 48 h, and 72 h post-exposure following the protocol of Lees et al. (2019) [47] with the addition of the 72 h timepoint to assess any residual toxicity. The assay was repeated using non-bloodfed mosquitoes (as described above) as the negative control. The use of non bloodfed mosquitoes was included to test whether any of the inhibitors had tarsal activity in the absence of a bloodmeal.

### Measuring the intrinsic activity of nitisinone through topical application immediately post-blood-feeding

The intrinsic activity of nitisinone was measured by topically applying a nitisinone solution to the dorsal thorax of bloodfed mosquitoes (Kisumu). Kisumu was selected as an insecticide-susceptible reference line to allow a direct comparison with the Lees et al. (2019) [47] dataset. Solutions of nitisinone were made by dissolving nitisinone in acetone to produce concentrations of 1%, 0.1%, 0.01%, 0.001% and 0.0001% (w/v). These solutions equate to 6, 0.6, 0.06, 0.006 and 0.0006 nmol/insect respectively. The concentrations were based on previous work used to screen insecticides against the Kisumu strain [47]. Permethrin (Merck Life Science, Gillingham, UK), at 1% in acetone, was used as the positive control and the carrier, acetone alone, was the negative control.

In groups of 60, mosquitoes were cold anesthetized on ice for 10 min. Once immobilized, the mosquitoes were oriented so their dorsal thorax was facing up. A 0.2 µl drop of solution was applied to the dorsal thorax using a blunt end Hamilton syringe (Merck Life Science, Gillingham, UK) (Fig. 1). This volume was selected on the basis of the closest achievable volume to the 0.25 µl used in Lees et al. (2019) [47] and was constrained by the capabilities of the syringe dispenser. After dosing, mosquitoes were transferred to a large fabric cage (Bugdorm-4M3030 Watkins and Doncaster, Leominster, UK) to recover. The process was repeated with another set of 60 mosquitoes/concentration to make two replicates totaling 120 mosquitoes per dose. Mortality was measured at 24 h and 48 h post-application.

### Determination of discriminating dose using the Centers for Disease Control and Prevention (CDC) bottle assay

To determine the discriminating dose of nitisinone, a modification of the WHO Bottle assay (Fig. 1) was adopted [48]. Selected concentrations were established through dose determination assays (data not shown) and were selected to be 0, 15, 18, 20, 21.5, 22.5, 23.5, 25, and 30 µg per bottle with a 30 µg per bottle permethrin as a control. Although WHO guidelines recommend 21.5 µg per bottle as the discriminating dose for *An. gambiae*, 30 µg was used here for consistency with standardized laboratory protocols. Nitisinone was dissolved in acetone at these concentrations and then added to 250 ml Wheaton Bottles (Fisher Scientific, Loughborough, UK). Bottles were coated following previous methods [47] and allowed to dry overnight before use for approximately 16 h. To conduct the assay, 25 bloodfed female Kisumu mosquitoes (fed within 1 h) were added to coated bottles and exposed for 1 h. Mortality was scored at 1 h and 24 h. Three replicates were performed per concentration and the assay was repeated twice to total three biological replicates.

### Measuring the efficacy of nitisinone against susceptible and insecticide-resistant *An. gambiae* s.l., *C. quinquefasciatus*, and *Ae. aegypti*

Using the glass plate bioassay previously described [47], a total of 3 replicates (biological) of 30 mosquitoes (10 mosquitoes per plate) were exposed to nitisinone-coated glass Petri dishes (radius 2.5 cm, area 19.635 cm^2^, SLS, Nottingham, UK). The assay was conducted in precisely the same fashion as for Kisumu. This assay compared the potency of nitisinone against bloodfed Kisumu, Tiassalé 13, VK7 2014, *C. quinquefasciatus*, and *Ae. aegypti.* Each species was tested separately. For all species except *Ae. aegypti*, three replicates were performed: *Ae. aegypti* had a single replicate.

### Statistical analysis

Graphs were constructed using GraphPad Prism (Version 9.4.1), except for the discriminating dose graph and probit analysis, which were constructed using PoloSuite 2.0 (LeOra Software). A two-way analysis of variance (ANOVA) (GraphPad) was used to assess the significance between datasets followed by Tukey’s multiple comparison test where applicable. A significance threshold of *P* < 0.05 was used throughout. Mortality was corrected using the Abbott’s Formula; if the negative control mortality was between 5% and 20%, a correction was made [49]. There was no correction if mortality was below 5% and replicates were discarded if mortality exceeded 20%.

### Image production

All images were captured using a Nikon VVA241K001 1 J5 Compact System Camera (20.8 Mp) either handheld or mounted onto a Stemi 305 Stereo Microscope 8x-40x (Leica). Images were cropped, de-speckled, noise corrected, and size adjusted using Adobe PhotoShop CS (24.7.0 release).

## Results

### Mosquitoes must ingest blood to activate HPPD inhibitor-associated killing after cuticular uptake

**Figure 2:**
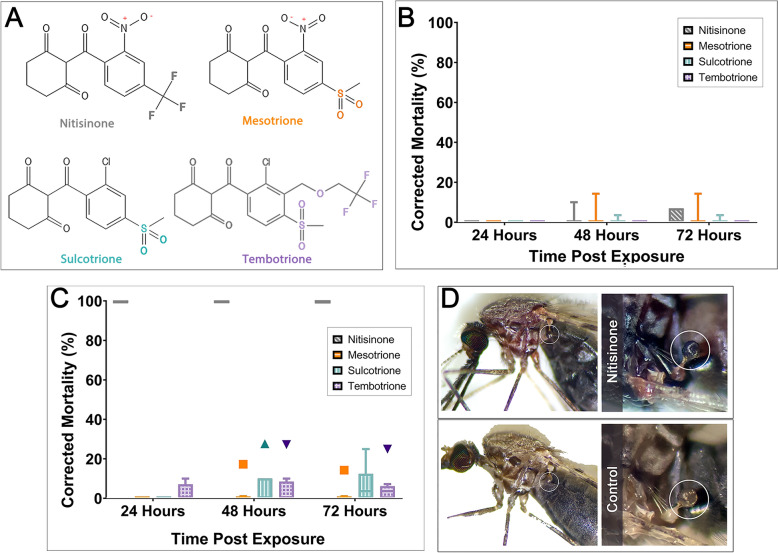
Comparison of mosquito mortality following tarsal contact with a high concentration of HPPD inhibitors (125 mg/m^2^). **A** The four β-triketones contain a triketone backbone and variable residues on the phenyl group. **B** Sugar-fed *An. gambiae* Kisumu tested on plates coated with the HPPD inhibitors: nitisinone (*n* = 93; grey), mesotrione (*n* = 92; orange), sulcotrione (*n* = 98; turquoise), and tembotrione (*n* = 95; purple); all concentrations were at 125 mg/m^2^. Time post-exposure is shown as 24 h, 48 h, and 72 h. **C** Bloodfed *An. gambiae* Kisumu survival upon tarsal exposure to HPPD inhibitors: nitisinone (*n* = 90; grey), mesotrione (*n* = 96; orange), sulcotrione (*n* = 93; turquoise), and tembotrione (*n* = 96; purple) 125 mg/m^2^. Time post-exposure is shown as 24 h, 48 h, and 72 h. **D** Exposure to nitisinone causes paralyzed mosquitoes to systemically darken. The halteres (shown within the white circles) are particularly noticeable

Sugar-fed, female *An. gambiae* (Kisumu) were tested on tarsal assays. Very low mortality was observed even at the highest dose tested (125 mg/m^2^) for all HPPD-inhibitors screened (Fig. 2A and B). The corrected mortality at 72 h post-exposure was nitisinone (5.78%), mesotrione (5.25%), sulcotrione (1.31%), and tembotrione (0%).

However, when mosquitoes were exposed to nitisinone before (Fig. S1) or after blood-feeding, nitisinone was the only inhibitor that was significantly mosquitocidal (*P* < 0.0001) (Fig. 2C) despite the highest inhibitor concentration at 125 mg/m^2^ used for all inhibitors. None of the other inhibitors reached the 80% mortality breakpoint (defined previously as a measure of adequate efficacy of a product [47]); even after 72 h post-exposure, sulcotrione (13.24%), tembotrione (11.06%), and mesotrione (5.35%) failed to kill mosquitoes. Of note, exposure to nitisinone caused paralysis in all mosquitoes and they darkened in color (Fig. 2D), in line with the darkening phenotype reported in tsetse treated with nitisinone [26].

### Topical application of nitisinone on *An. gambiae* shows high intrinsic activity

**Figure 3:**
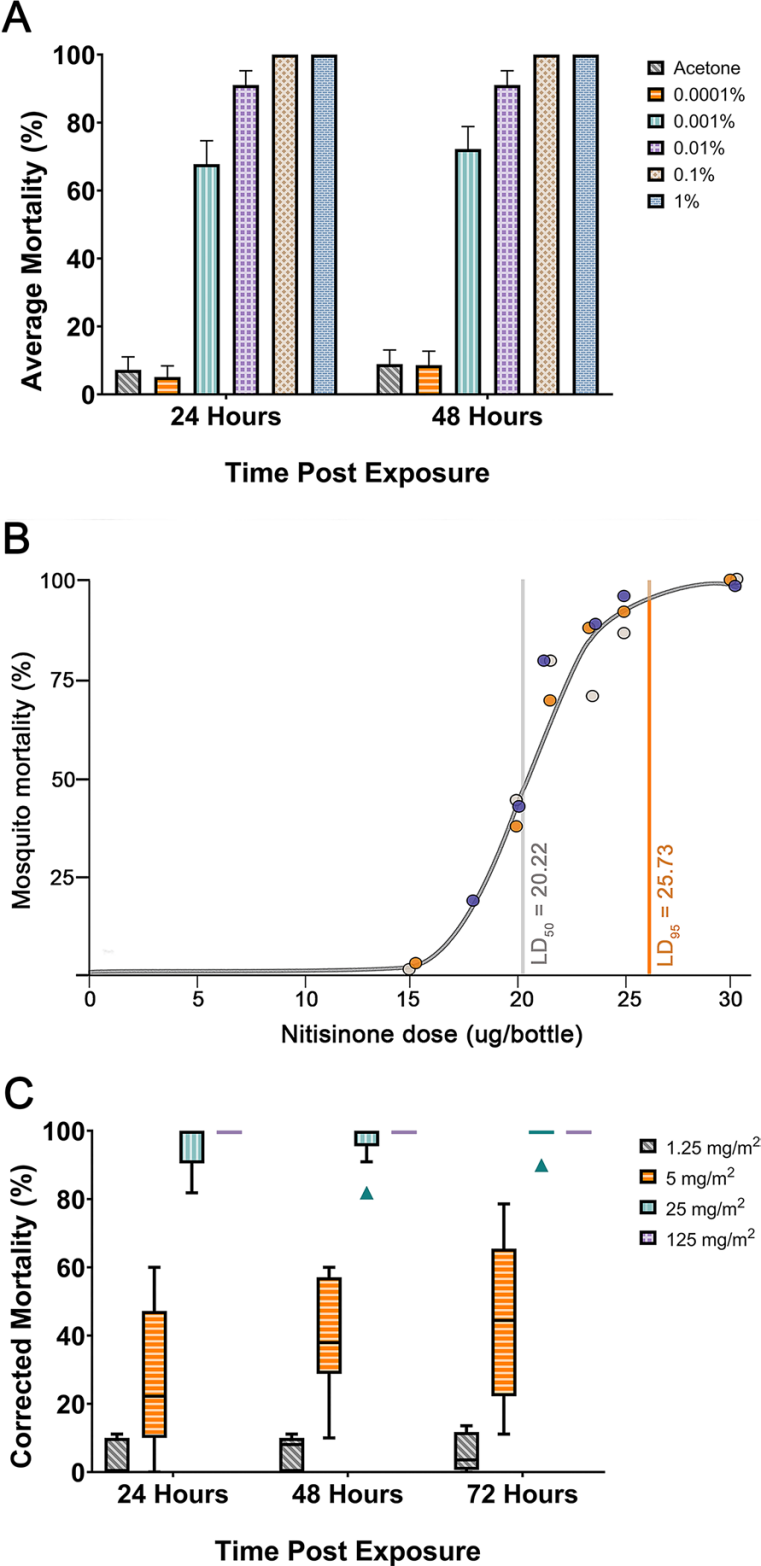
Mortality of female *An. gambiae* s.s. Kisumu to topical, bottle, and tarsal assays with nitisinone. **A** Topical assay using bloodfed *An. gambiae* Kisumu tested against a dilution series of nitisinone (three replicates, *n* = 180). **B** Bottle assay testing several concentrations of nitisinone. Rep 1 (grey), Rep 2 (purple), Rep 3 (orange). LD_50_ (grey vertical line) is the dose of nitisinone killing 50%. LD_95_ (orange vertical line) is 95% of the population is killed at this dose. **C** Tarsal assay on bloodfed mosquitoes exposed to four different nitisinone concentrations (1.25–125 mg/m^2^)

A single volume of 0.2 µl of each nitisinone dilution was topically applied to the thorax of bloodfed mosquitoes and mortality was scored over 48 h. Over 50% killing at concentrations of 0.001% nitisinone (0.01 mg/ml, representing a dose of 2 ng/mosquito) and above was observed (Fig. 3A). Only 0.0001% nitisinone (0.2 ng/mosquito) was not significantly different from the negative control. The breakpoint of 80% was met with the 0.01% (20 ng/mosquito) solution within 24 h. Collectively, this shows evidence of the high intrinsic activity (inherent potency) of the cuticular exposure of nitisinone in bloodfed mosquitoes. This performance compares favorably with the results reported in Table 1 of Lees et al. (2019) [47], where many candidate compounds screened in topical assays failed to reach the mortality thresholds met by nitisinone.

### Discriminating dose of nitisinone

As nitisinone was the only mosquitocidal HPPD inhibitor in the tarsal and topical bioassays, we continued to follow the testing cascade [47] using the WHO Bottle Assay to establish a discriminating dose as a measure of potency (Fig. 3B). Bloodfed *An. gambiae* Kisumu were exposed to nitisinone-coated bottles in a modified CDC Bottle Assay [48] and the dose–response graph is shown (Fig. 3B). The LD_**50**_ of nitisinone was calculated at 20.22 µg/bottle (95% CI 19.55–20.69), which is equivalent to 0.72 mg/m^2^. The LD_**95**_ was calculated at 25.73 µg/bottle (95% CI 24.65–27.37), which is equivalent to 0.92 mg/m^2^. The discriminating dose is calculated as three times the LD_**95**_ and is therefore 77.19 µg/bottle, which is equivalent to 2.76 mg/m^2^.

### Insecticide-resistant strains of *An. gambiae* are killed by tarsal uptake of nitisinone

**Figure 4:**
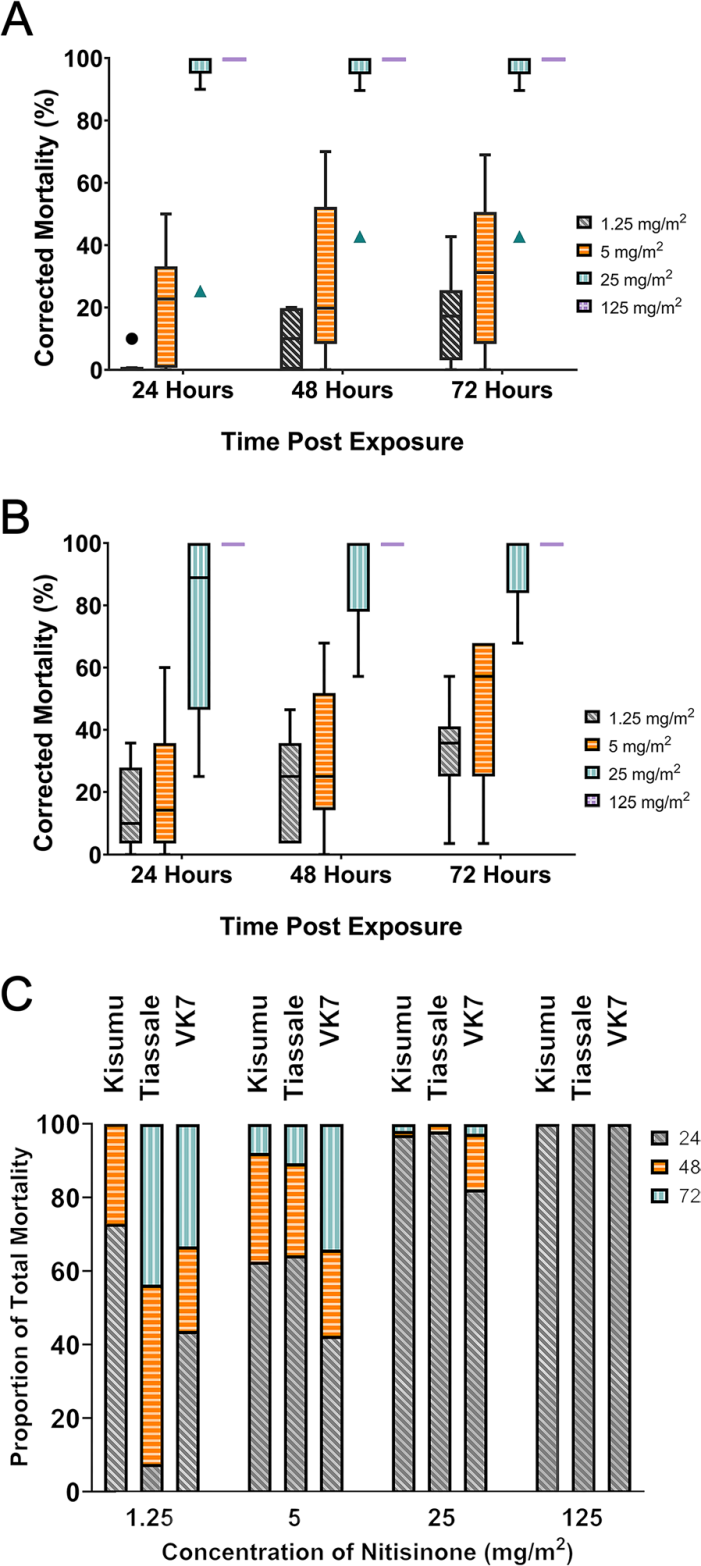
Tarsal assays testing nitisinone efficacy against insecticide resistant *An. gambiae* s.l. Tiassalé 13 (**A**) and VK7 2014 (**B**). Time taken for mortality to occur in each species of *An. gambiae* (0–72 h) (**C**). Speed of killing as a proportion of total mortality for each strain of *An. gambiae* tested at each concentration

Different strains of insecticide-resistant and susceptible *Anopheline* mosquitoes were tested for susceptibility to nitisinone upon tarsal contact. These resistant mosquito strains were selected because of their well-documented resistance mechanisms to several insecticide classes and carefully documented GLP rearing conditions, including maintaining under insecticide selection pressure and genotyping for contamination (Table S2). *An. gambiae* Kisumu (susceptible, Fig. 3C), Tiassalé 13 (resistant, Fig. 4A), and *An. coluzzii* VK7 2014 (resistant, Fig. 4B) all died within 24 h of exposure to nitisinone at 125 mg/m^2^. At a fivefold lower dose, 25 mg/m^2^, the killing effect was slightly reduced (insignificant) although it remained above the 80% threshold by 72 h post-exposure: Kisumu (98.9%), Tiassalé 13 (92.5%), and VK7 2014 (92.9%). At lower nitisinone doses (5 mg/m^2^ and 1.25 mg/m^2^) the mortality for all timepoints was below the 80% threshold, suggesting the cutoff for this assay was 25 mg/m^2^ for all strains tested. At 72 h there was no significant difference between the strains at any concentration except at 1.25 mg/m^2^ between Kisumu (which was significantly lower (*P* = 0.003) than VK7 2014). Comparing how long it took for the mosquitoes to die post-exposure, there is no clear pattern (Fig. 4C). However, at the lowest dose, Kisumu reaches its maximum mortality quicker than Tiassalé 13, which is slower to do so at 25 mg/m^2^. It seems that VK7 2014 is slower to reach maximum killing at 5 mg/m^2^ and 25 mg/m^2^.

### Insecticide-resistant strains of *C. quinquefasciatus* and insecticide susceptible strains of *Ae. aegypti* are killed by the tarsal uptake of nitisinone

**Figure 5:**
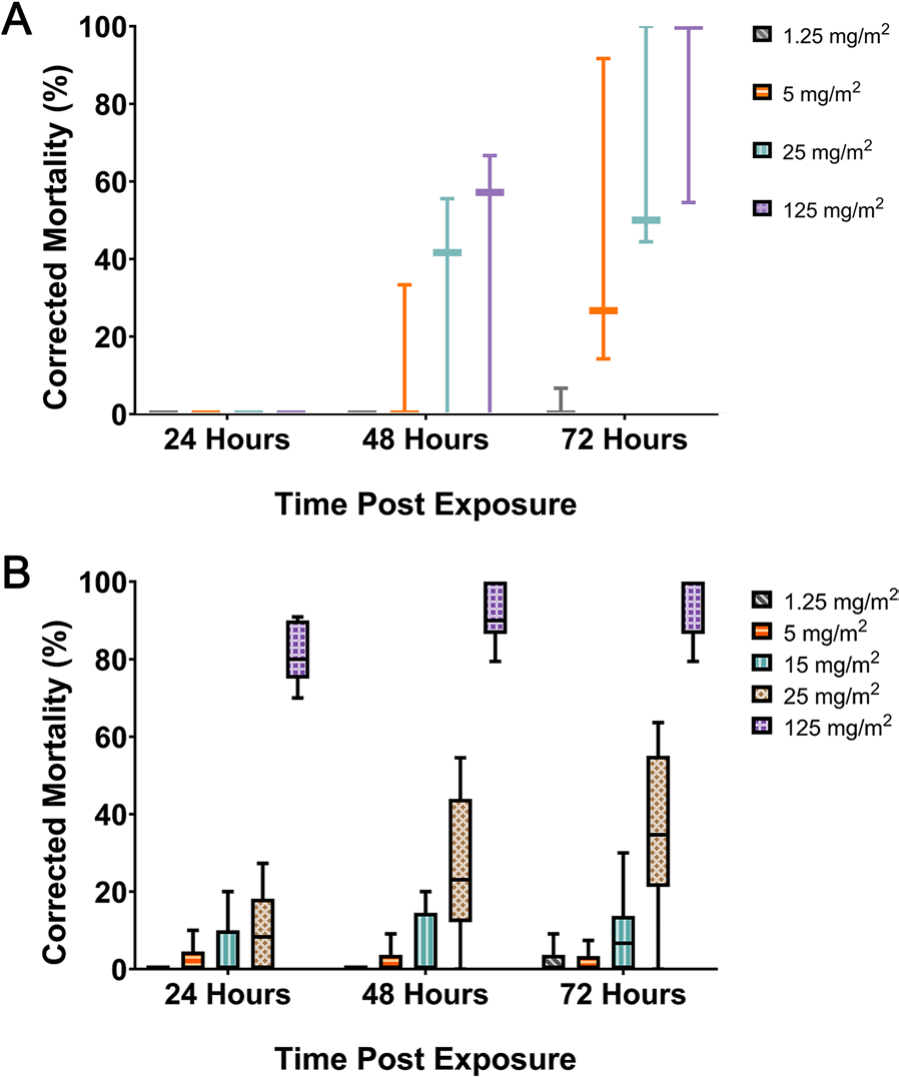
*Ae. aegypti* (**A**) and *C. quinquefasciatus* (**B**) susceptibility profiles to tarsal contact with nitisinone. **A** Nitisinone concentrations tested: 1.25 mg/m^2^ (grey), 5 mg/m^2^ (orange), 25 mg/m^2^ (turquoise), and 125 mg/m^2^ (purple). **B** 1.25 mg/m^2^ (grey), 5 mg/m^2^ (orange), 15 mg/m^2^ (turquoise), 25 mg/m^2^ (brown), and 125 mg/m^2^ (purple). Three biological replicates represent *n* = 90 per dose

**Figure 6:**
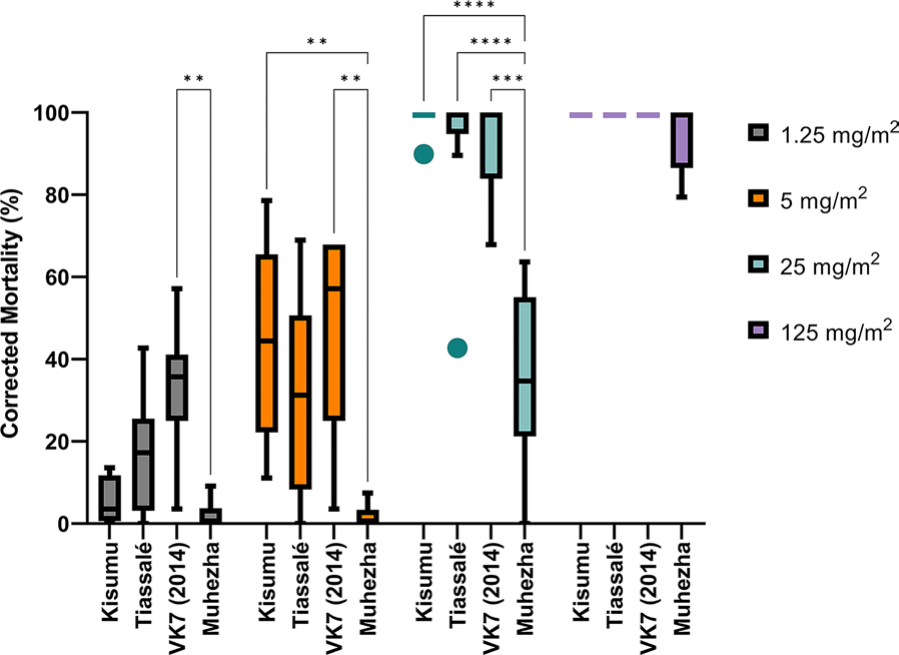
The comparative mortality of four mosquito strains 72 h post-tarsal exposure to nitisinone. Kisumu (insecticide-susceptible *An. gambiae*), Tiassalé 13 (insecticide-resistant *An. gambiae*), VK7 2014 (insecticide-resistant *An. coluzzii*), Muheza (insecticide-resistant *C. quinquefasciatus*) (*x*-axis). Three biological replicates for each group with an average of 90 mosquitoes were used per replicate (or used for each assay). Nitisinone concentrations were: 1.25 mg/m^2^ (grey), 5 mg/m^2^ (orange), 25 mg/m^2^ (turquoise), and 125 mg/m^2^ (purple)

To confirm that the tarsal exposure of nitisinone had broad activity against other mosquito species, the insecticide-resistant strain of* C. quinquefasciatus* Muheza (Fig. 5A) and the insecticide-susceptible *Ae. aegypti* New Orleans strain (Fig. 5B) were exposed to glass tarsal plates coated with nitisinone. For Muheza at 125 mg/m^2^, there was a breakpoint killing of 81.6% at 24 h, which increased to 94.7% by 72 h. This killing effect was slightly lower, though not significant, compared with the 100% mortality observed in the *Anopheles* strains (Fig. 6). At 25 mg/m^2^, the killing effect was significantly reduced (< 0.0001) to 35.8% by 72 h. Mortality decreased further at 15 mg/m^2^ (7.9% at 72 h), 5 mg/m^2^ (1.6% at 72 h), and 1.25 mg/m^2^ (1.8% at 72 h), indicating a potentially higher innate resistance to lower concentrations of nitisinone. The reduction in killing efficacy by 72 h, compared with the *Anopheles* strains, was significant (*P*-values ranging from 0.03 to < 0.0001) across all comparable doses: 25 mg/m^2^ and 5 mg/m^2^ (15 mg/m^2^ was not tested in *Anopheles*), and at 1.25 mg/m^2^ for VK7 2014 and Tiassalé 13 (though not significant for Kisumu).

For *Ae. aegypti* New Orleans (Fig. 5B), the potency of nitisinone was reduced compared with both *Anopheles* and *Culex*. The breakpoint killing at 125 mg/m^2^ was not reached until 48 h (84.8%), rising to 97.0% by 72 h. No other concentration passed the breakpoint by 72 h: 25 mg/m^2^ (77.8%), 5 mg/m^2^ (60.3%), and 1.25 mg/m^2^ (6.7%).

However, despite mortality differences at the highest dose between New Orleans and Muheza, by 24 h, all other concentrations were more potent in New Orleans (susceptible) than Muheza (resistant).

## Discussion

In the pursuit of innovative vector control strategies, a promising avenue to identify new insecticidal compounds is to extend the search beyond conventional neurological and detoxification gene targets to include insect blood-feeding mechanisms. Previously, nitisinone was shown to be either toxic when ingested by blood-feeding insects or when absorbed through the cuticle via topical application (solvent assisted) [26–28, 50]. Adding to previous results that nitisinone induced mortality in tsetse and triatomines, we provide additional evidence that inhibiting 4-hydroxyphenylpyruvate dioxygenase (HPPD) constitutes a viable approach for mosquito control via cuticular uptake. The four β-triketone HPPD inhibitors we investigated were originally selected on commercial availability and potency on plant targets, or in the case of nitisinone, Food and Drug Administration (FDA) approval as a human drug [23, 25]. We have shown that nitisinone is the only HPPD inhibitor with contact-based mosquitocidal effect. The lack of significant toxicity of mesotrione, sulcotrione, and tembotrione was unexpected, but it might reflect differences in the cuticular penetrability among these chemistries. Furthermore, nitisinone killed mosquito strains resistant to several insecticide classes, providing evidence of its potential to control the growing problem of insecticide-resistant mosquitoes that are undermining vector-based disease control efforts. Using the glass plate tarsal assay, there was no difference in mortality at 5, 25, and 125 mg/m^2^ nitisinone between the insecticide-susceptible Kisumu strain and the insecticide-resistant Tiassalé 13 and VK7 2014 strains. This is not surprising, as tyrosine metabolism is essential to hematophagous insects and therefore its inhibition should be lethal to blood-feeding arthropods [50]. We predicted that VK7 2014, and to some extent Tiassalé 13, would show less mortality than Kisumu because of cuticular changes to the tarsi that slow uptake of insecticides (i.e., thickness of cuticle) [51, 52]. However, this was not observed, thus indicating tarsi remain equally permeable to nitisinone. It remains to be determined whether the sensory appendage protein 2, which has been shown to be compound-specific in resistant mosquitoes, potentially mediates nitisinone uptake across the cuticle rather than a generic cuticular thickening [53]. Despite its delayed mortality profile, nitisinone could still offer operational value in integrated vector control by acting before oviposition or reducing fecundity in bloodfed, disease-transmitting females.

Incorporating data from several types of assays can enhance the robustness of insecticide efficacy evaluations. However, it is essential to acknowledge that the topical application assay is the least representative of field conditions among the three methods discussed. The direct application of insecticide onto the mosquito thorax using an aqueous solution does not mimic a typical environmental exposure scenario for *An. gambiae* s.l. [47], although it can give a rough indication of *Anopheline* susceptibility to a compound. Although the glass plate and bottle assays both measure bioefficacy via tarsal contact, their results are not directly comparable. Differences in exposure duration and surface coating significantly influence the mortality rates observed in each assay, thus highlighting the importance of assay selection to accurately assess insecticide efficacy.

Insecticide residual spraying (IRS) exploits the mosquito behavior to rest post-blood-feeding, and consequently, adsorb insecticide upon contact with an insecticide-covered surface. The efficacy of IRS can be significantly reduced by insecticide degradation, suboptimal coverage, and modifications to sprayed surfaces such as wall washing post-treatment. These issues cause two problems: (1) mosquito survival post-exposure to a nonlethal dose and (2) while resistance is primarily driven by lethal selection, repeated sublethal exposures may contribute to resistance development by allowing survival of partially resistant individuals and maintaining alleles associated with reduced susceptibility [54]. As we used bloodfed mosquitoes rather than the industry standard of sugar-fed, direct comparisons with previous published data are not possible. However, comparing discriminating dose (DD) and dose response curve shape for nitisinone with data generated for other compounds [47] is encouraging. The discriminating dose combines a fixed exposure time, and the amount of insecticide coated inside a glass bottle with the uptake depending on the time of actual tarsal contacts. On the basis of these results, the potency of nitisinone is greater than clothianidin, spinetoram, metaflumizone, and dinotefuran [47], which makes it attractive for further optimization as novel IRS formulations. Considering the gradient of the dose–response curve (roughly determined by calculating the gradient from the LC_95_ and LC_50_ derived from Fig. 3), nitisinone has the steepest curve, indicative of high potency. This reflects previous nitisinone results in both blood-feeding and topical application assays in another dipteran disease vector, *Glossina morsitans morsitans* [26]. We briefly tested the efficacy of nitisinone (using the glass plate assay) by exposing Kisumu (Fig. S1A) or New Orleans (Fig. S1B) before blood-feeding. Nitisinone is still active tarsally and this models a mosquito landing on a sprayed wall before taking a blood meal, which warrants further investigation. It is possible that nitisinone’s efficacy (and perhaps that of other HPPD inhibitors) through tarsal contact could be increased by combining adjuvants such as rapeseed oil methyl esters (RME), as described with other insecticides [44, 55]. By testing the exposure to RME pre-blood feed with Kisumu (Fig. S2), we showed that combining an adjuvant such as RME significantly increased mosquito mortality at the 5 mg/m^2^ concentration.

There are compelling reasons to formulate nitisinone into an IRS: (1) nitisinone targets bloodfed (naturally more insecticide tolerant) mosquitoes resting on sprayed surfaces indoors, suggesting a novel mechanism of action that could potentially overcome increased insecticide-resistance [4]; (2) it is stable under a range of environmental conditions such as UV radiation, temperature, and pH [26, 56]; (3) it is unlikely to be immediately metabolized by P450 enzymes, although resistance through P450 metabolism could slowly evolve with selective pressure [26]; and (4) its relatively low water solubility and presumed high lipophilicity may enhance cuticular penetration, which could explain its superior contact-based activity compared with other HPPD inhibitors tested (Table S1).

The killing kinetics of unformulated nitisinone across the different insecticide-resistant mosquito strains is intriguing. Strain VK7 2014 may die slower because of cuticular thickening, reduced blood meal size or speed of bloodmeal digestion, which we did not examine. The lower toxicity of nitisinone with the insecticide-resistant *Culex* strain, Muheza, indicates that a higher concentration (between 25 and 125 mg/m^2^) needs further investigation. Additionally, as with *Culex, Aedes* showed reduced susceptibility to nitisinone compared with *Anopheles*, which may indicate physiological differences between the two species in blood-feeding and digestion rates [27]. These differences highlight the importance of understanding species-specific traits when evaluating bloodmeal-activated insecticides. Despite its bloodmeal dependency and delayed killing profile, nitisinone may still be operationally useful, as it is likely to either act before oviposition can take place or reduce overall fecundity. Given its novel mode of action targeting the tyrosine degradation pathway via inhibition of 4-hydroxyphenylpyruvate dioxygenase (HPPD) enzyme, nitisinone offers promise as part of an integrated vector management strategy. However, the potential for resistance evolution through target-site mutation or metabolic adaptation must be considered, and further research is ongoing to investigate these mechanisms.

## Conclusions

Our results show that nitisinone kills bloodfed mosquitoes via tarsal contact while mesotrione, sulcotrione, and tembotrione do not. This killing did not discriminate between mosquito strains susceptible or highly resistant to other insecticide classes (including pyrethroids, organochlorides, and potentially carbamates). Moreover, the mosquitocidal efficacy of nitisinone via cuticular absorption extends beyond *Anopheline* species, as demonstrated by its effectiveness against *C. quinquefasciatus* and *Ae. aegypti*. Our data support further investigation into optimizing nitisinone uptake, potentially by using chemistries to enhance cuticular absorption or by formulating nitisinone with adjuvants. With its novel mechanism of action, nitisinone can exploit the blood-feeding behaviors of female mosquitoes. This makes it a promising candidate for innovative indoor residual sprays and long-lasting insecticidal nets, particularly in regions where traditional mosquito control methods are being undermined by the rapid emergence of pyrethroid resistance.

## Supplementary Information

Additional file 1.

Additional file 2.

Additional file 3.

Additional file 4.

## Data Availability

Data supporting the main conclusions of this study are included in the manuscript.

## Abbreviations

HPPD: 4-Hydroxyphenylpyruvate dioxygenase
CDC: Centers for Disease Control and Prevention
IRAC: Insecticide Resistance Action Committee
LLIN: Long-lasting insecticidal net
IRS: Indoor residual spray
WHO: World Health Organization
DD: Discriminating dose
LD: Lethal dose
LSTM: Liverpool School of Tropical Medicine
LITE: Liverpool Insect Testing Establishment
DDT: Dichlorodiphenyltrichloroethane
FDA: Food and Drug Administration
LC: Lethal concentration
RME: Rapeseed oil methyl esters
