# Supplementary figures and images for: The β-triketone, nitisinone, kills insecticide-resistant mosquitoes through cuticular uptake
Source: Parasit Vectors. 2025 Jul 31;18:316. doi: 10.1186/s13071-025-06939-0 (PMC12315382; doi:10.1186/s13071-025-06939-0)

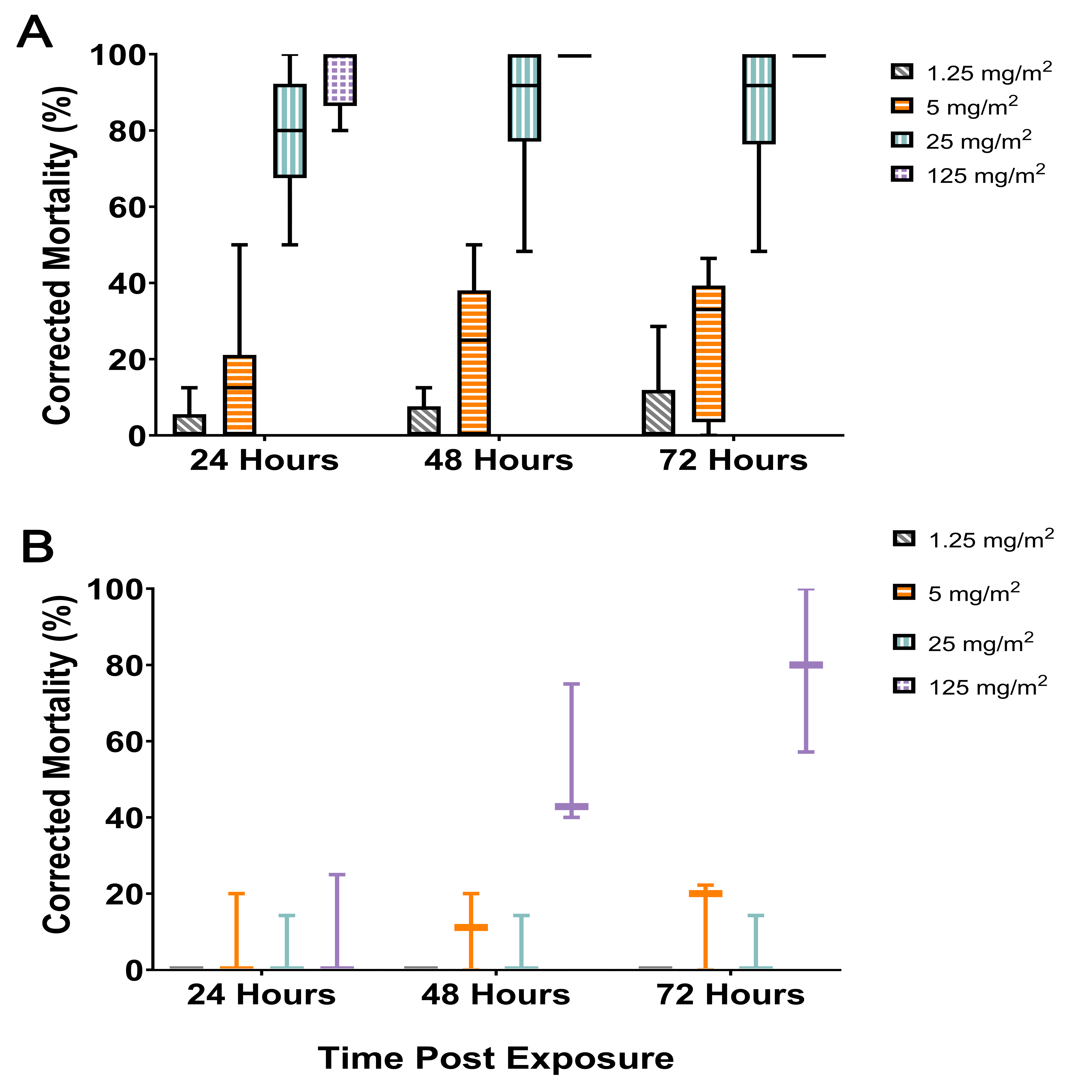

Supplement: Supplementary file 1 — Additional file 1. [file 13071_2025_6939_MOESM1_ESM.tif]

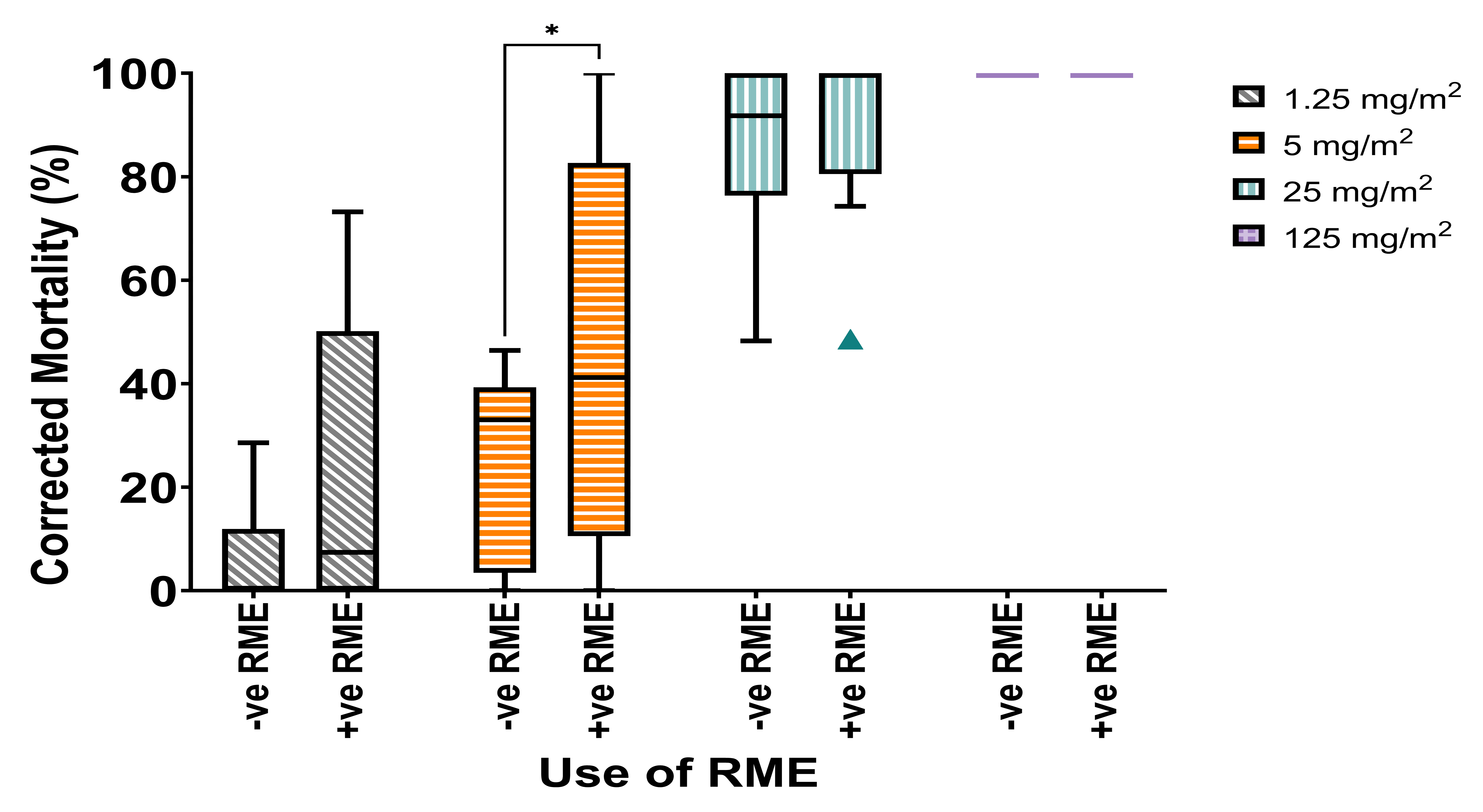

Supplement: Supplementary file 2 — Additional file 2. [file 13071_2025_6939_MOESM2_ESM.tif]
