# Supplementary material for: The β-triketone, nitisinone, kills insecticide-resistant mosquitoes through cuticular uptake
Source: Parasit Vectors. 2025 Jul 31;18:316. doi: 10.1186/s13071-025-06939-0 (PMC12315382; doi:10.1186/s13071-025-06939-0)
Supplement: Supplementary file 3 — Additional file 3. [file 13071_2025_6939_MOESM3_ESM.docx]

**Table S1.** **HPPD inhibitors used within this study.** Compounds are given with their manufacturer and usage. Chemical and Toxicological information shown.

| **Compound** | **Manufacturer** | **Use as Herbicide or Medicine** | **Solubility - In water at 20 °C (mg/L:)** | **Degradation Point** | **Mammals - Acute oral LD₅₀ (mg/kg)** | **Soil degradation (days) (aerobic)**  **DT₅₀ (typical)** |
| --- | --- | --- | --- | --- | --- | --- |
| Nitisinone | Swedish Orphan Biovitrum Ltd | Hereditary tyrosinaemia type I  Alkaptonuria | 8.11 | n/a | n/a | n/a |
| Mesotrione | Greencrop  Syngenta  Chemsource | Grass and broad-leaved weeds | 1500 | 166 | >5000 | 19.6 |
| Sulcotrione | Syngenta | Grass and broad-leaved weeds | 165 | 170 | >5000 | 25 |
| Tembotrione | Bayer CropScience | Broad-leaved and grassy weeds | 71000 | 150 | >2500 | 14.5 |
