# Supplementary material for: The β-triketone, nitisinone, kills insecticide-resistant mosquitoes through cuticular uptake
Source: Parasit Vectors. 2025 Jul 31;18:316. doi: 10.1186/s13071-025-06939-0 (PMC12315382; doi:10.1186/s13071-025-06939-0)
Supplement: Supplementary file 4 — Additional file 4. [file 13071_2025_6939_MOESM4_ESM.docx]

**Table S2. Insecticide resistance status of each mosquito strain used in this study.** Status was determined through WHO Tube Assay and correct as of 2024. Insecticide classes are Pyrethroids (PY), organochlorides (OC), carbamates (CB) and organophosphates (OP).

| **Species** | **Strain** | **Resistance Status** |
| --- | --- | --- |
| *Anopheles gambiae* | Kisumu | Permethrin (PY): susceptible |
|  |  | Deltamethrin (PY): susceptible |
|  |  | Alpha-cypermethrin (PY): susceptible |
|  |  | DDT (OC): low levels of resistance detected |
|  |  | Dieldrin (OC): susceptible |
|  |  | Propoxur (CB): susceptible |
|  |  | Fenitrothion (OP): susceptible |
|  | Tiassalé 13 | Permethrin (PY): resistant |
|  |  | Deltamethrin (PY): resistant |
|  |  | Alpha-cypermethrin (PY): resistant |
|  |  | DDT (OC): resistant |
|  |  | Dieldrin (OC): resistant |
|  |  | Propoxur (CB): possible resistance |
|  |  | Fenitrothion (OP): susceptible |
|  | VK7 2014 | Permethrin (PY): resistant |
|  |  | Deltamethrin (PY): resistant |
|  |  | Alpha-cypermethrin (PY): resistant |
|  |  | DDT (OC): resistant |
|  |  | Dieldrin (OC): susceptible |
|  |  | Propoxur (CB): susceptible |
|  |  | Fenitrothion (OP): susceptible |
| *Aedes aegypti* | New Orleans | Permethrin (PY): susceptible |
|  |  | Deltamethrin (PY): susceptible |
|  |  | Alpha-cypermethrin (PY): susceptible |
|  |  | DDT (OC): susceptible |
|  |  | Dieldrin (OC): susceptible |
|  |  | Propoxur (CB): susceptible |
|  |  | Fenitrothion (OP): susceptible |
| *Culex quinquefasciatus* | Muheza | Permethrin (PY): resistant |
|  |  | Deltamethrin (PY): resistant |
|  |  | Alpha-cypermethrin (PY): resistant |
|  |  | DDT (OC): resistant |
|  |  | Dieldrin (OC): susceptible |
|  |  | Propoxur (CB): possible resistance |
|  |  | Fenitrothion (OP): susceptible |
